# Changes in the vaginal microbiome during pregnancy and the postpartum period in South African women: a longitudinal study

**DOI:** 10.21203/rs.3.rs-2617869/v1

**Published:** 2023-04-19

**Authors:** Katherine Li, Fan Li, Heather Jaspan, Dorothy Nyemba, Landon Myer, Grace Aldrovandi, Dvora Joseph-Davey

**Affiliations:** UCLA Health System: University of California Los Angeles Health System; UCLA Medical School: University of California Los Angeles David Geffen School of Medicine; University of Cape Town Department of Pathology; University of Cape Town Faculty of Health Sciences; University of Cape Town Faculty of Health Sciences; UCLA Medical School: University of California Los Angeles David Geffen School of Medicine; University of Cape Town Faculty of Health Sciences

**Keywords:** vaginal microbiome, microbiome in pregnancy, South Africa

## Abstract

African women have more diverse vaginal microbiota than women of European descent, and there is interest in the impact of this diversity on maternal health, including HIV and STI acquisition. We characterized the vaginal microbiota in a cohort of women ≥ 18 years with and without HIV in a longitudinal cohort over two visits during pregnancy and one visit postpartum. At each visit we obtained HIV testing and self-collected vaginal swabs for point of care testing for STIs and microbiome sequencing. We categorized microbial communities and evaluated changes over pregnancy and associations with HIV status and STI diagnosis. Across 242 women (mean age 29,44% living with HIV, 33% diagnosed with STIs), we identified four main community state types (CSTs): two lactobacillus-dominant CSTs (dominated by *Lactobacillus crispatus* and *Lactobacillus iners* respectively) and two diverse, non-lactobacillus-dominant CSTs (one dominated by *Gardnerella vaginalis* and one by other facultative anaerobes). From first antenatal visit to third trimester (24–36 weeks gestation), 60% of women in the *Gardnerella*-dominant CST shifted to *Lactobacillus*-dominan CSTs. From third trimester to postpartum (mean 17 days post-delivery), 80% of women in *Lactobacillus*-dominant CSTs shifted to non-lactobacillus-dominant CSTs with a large proportion in the facultative anaerobe-dominant CST. Microbial composition differed by STI diagnosis (PERMANOVA R^2^ = 0.002, p = 0.004), and women diagnosed with an STI were more likely to be categorized with L. *iners*-dominant or *Gardnerella*-dominant CSTs. Overall we found a shift toward lactobacillus dominance during pregnancy, and the emergence of a distinct, highly diverse anaerobe-dominant microbiome population in the postpartum period.

## Introduction

Sub-Saharan Africa faces significant challenges in maternal and infant health. In 2017, 66% of all maternal deaths worldwide occurred in sub-Saharan Africa [1], and there were an estimated 50 infant deaths for every 1000 live births in 2020 [2]. Many of these adverse birth outcomes are caused by preventable or curable infectious diseases. HIV accounts for 24–50% of pregnancy-related mortality in areas of high prevalence, and is a leading cause of death of reproductive-aged cisgender women [3, 4]. The risk of HIV acquisition is increased during pregnancy and postpartum period, which then also increases the risk of vertical transmission [5]. HIV incidence remains high in pregnant and breastfeeding women in South Africa: in a recent study in Cape Town, postpartum HIV incidence was 1.86/100 person-years (95% CI 0.88–3.89), and incidence was highest during the first 6 months postpartum (2.71/100 person-years, 95% CI 1.13–6.51) [6]. Sub-Saharan Africa also has the highest incidence rate of bacterial STIs in the world, with 60 million new infections every year [7]. Untreated STIs in pregnancy lead to adverse pregnancy outcomes including preterm birth, stillbirth, and infant death [8]. In South Africa specifically, the prevalence of bacterial STIs during pregnancy ranges from 30–40% [9, 10].

There is growing interest in the role of the vaginal microbiome in the health of reproductive-aged women and their infants, particularly with respect to HIV and STI acquisition during pregnancy. Studies from North America and Europe have shown that a healthy vaginal microbiota is dominated by lactobacillus species, including *Lactobacillus crispatus, L. gasseri*, and L. *jensenii* [11], which modulate vaginal pH predominantly through production of D-lactic acid [12]. Conversely, microbiomes dominated by facultative anaerobes including *Gardnerella, Prevotella, Atopobium spp.* and others, are linked to bacterial vaginosis (BV). BV and anaerobe-dominant vaginal states may increase the risk of HIV and STI acquisition [13-16] and have been shown to be associated with adverse pregnancy outcomes including preterm birth [17, 18].

Subsequent work has demonstrated that the vaginal microbiome varies significantly with geography and ethnicity [11, 19]. African women and women of African descent have vaginal microbiota with far higher levels of non-lactobacillus anaerobes, including *Gardnerella, Prevotella*, and other BV-associated species [13, 20, 21]. They also have higher relative abundance of *Lactobacillus iners* which is more likely to coexist with rather than inhibit the growth of anaerobic bacteria [22, 23]. Observational studies have shown that differences in microbiome composition may increase the risk of acquiring HIV and STIs [15, 23, 24], and some studies have suggested that this may contribute to the disproportionately high burden of HIV and STIs among African women [13, 25, 26]. However, many *L. iners* and anaerobe-dominant profiles also occur in healthy African women and may represent normal variants that behave differently from lactobacillus-dominant profiles [20, 24, 27]. There is a need to characterize the vaginal microbiome in women of African descent to better understand what constitutes normal and identify patterns associated with risk of disease acquisition.

Pregnancy is a normal physiologic state that both influences and is influenced by the composition of the vaginal microbiome. Over the course of pregnancy, there is a gradual increase in lactobacillus species (including *L. iners*) and decreased microbial diversity, followed by a rapid increase in diversity and growth of anaerobic species in the postpartum period [17, 19, 21, 24, 28-30]. This is thought to be mediated by estrogen, which promotes glycogen deposition in the vaginal epithelium and supports lactobacillus proliferation [30, 31]. Given the associations between lactobacillus abundance and an optimal microbiome, studies have suggested that pregnancy induces a favorable change in the microbiome to prevent maternal genital infection and adverse birth outcomes [19]. Conversely, the rapid increase in bacterial diversity in the postpartum period [28, 30] may contribute to increased HIV risk during the postpartum period. Shifts in microbial composition during pregnancy may be particularly pronounced in women of African descent, as they have more diverse microbiome profiles in general [19]. Several cross-sectional studies performed in African countries have shown that pregnant women with HIV have more anaerobe-predominant microbiome profiles [14, 23, 27, 32], and a study from Kenya described species differences in pregnant women with *Chlamydia trachomatis* (CT) or *Trichomonas vaginalis* (TV) infection compared to uninfected women. However, to our knowledge there are few longitudinal studies of the vaginal microbiome in pregnant African women, and it is not known whether these microbiome transitions during pregnancy and postpartum have any impact on maternal and neonatal outcomes.

This study aims to characterize the vaginal microbiome over the course of pregnancy and in the immediate postpartum period in a cohort of women in Cape Town, South Africa. We also evaluated associations of microbial composition with HIV serostatus and STI diagnosis across the peripartum period.

## Methods

### Recruitment and Visit procedures

The STI in Pregnancy (STIP) study was conducted at a public sector antenatal clinic (ANC) in Cape Town, South Africa, as described previously [10]. Briefly, from November 2017 to July 2018, we enrolled pregnant women ≥ 18 years of age with and without HIV presenting to the ANC for prenatal care. Gestational age was estimated based on the date of the last menstrual period. Women participated in two visits over the course of their pregnancy and one in early postpartum: first visit to the ANC (Visit A), third trimester visit (Visit B), and one visit which occurred 7–10 days postpartum (Visit PPt). Women who presented for Visit A at gestational age > 28 weeks did not have a Visit B.

### Data collection

At each study visit, a trained study counselor collected data from a study survey on demographics, sexual behavior, health data (including HIV status and treatment), and any symptoms of STIs. Each woman then self-collected vulvovaginal swabs using Xpert^®^ CT/NG Vaginal/Endocervical Specimen Collection kits (Cepheid, Sunnyvale, CA). These swabs underwent on-site Xpert nucleic acid amplification testing for *Neisseria gonorrhea* (NG) and *Chlamydia trachomatis* (CT), and Xpert TV assay for *Trichomonas vaginalis* (Cepheid). Afterwards, swabs were sent to UCLA for microbiome analysis. Women were given same-day results for CT, NG, and TV testing if results were available before they left the clinic. Women with a positive STI test result based on the Xpert^®^ result or who reported symptoms and did not report previously receiving treatment for their STI at the same visit were given treatment in accordance with South African National guidelines [33]. CT infections were treated with 1 g azithromycin orally via directly observed therapy, NG with an intramuscular injection of 250 mg ceftriaxone plus 1 g azithromycin orally (or 2 g azithromycin in case of significant penicillin allergy), and TV with 400 mg metronidazole orally every twelve hours for seven days. Women who presented with symptoms including dysuria, unusual vaginal discharge, or vaginal itching were given all three antibiotics as syndromic treatment [33]. As per the national STI guidelines, women were given counselling, provided with condoms and partner notification/referral letter [33].

At enrollment, pregnant women with unknown or negative HIV serostatus were tested for HIV according to the South African National testing guidelines [33]. These women received a Toyo^®^ Anti-HIV ½ rapid assay, and those who tested positive received a confirmatory Determine^™^ HIV Ag/Ab Combo rapid test (Abbott, Chicago, IL). Women with known HIV reported whether they were taking antiretroviral therapy (ART), and their information was cross-matched with the national database to ascertain their viral load at their most recent Visit. Women who tested negative for HIV received repeat rapid HIV testing at each subsequent visit. At the postpartum visit, women were asked about their delivery details and infant outcomes, and this was verified against their antenatal clinical records. World Health Organization guidelines were used to categorize adverse pregnancy and birth outcomes [34].

### Microbiome profiling

Profiling of the bacterial microbiome of the collected vaginal swab samples was performed by sequencing of the V4 (515F/806R) region of the 16S rRNA gene as previously described [35]. Briefly, samples were transferred to Lysing Matrix E tubes (MP Biomedicals, Burlingame, CA, USA) with RLT lysis buffer (Qiagen, Hilden, Germany) and bead-beated on a TissueLyser (Qiagen). Following manufacturer protocol, AllPrep DNA/RNA/Protein kit (Qiagen) was used to extract DNA. In addition to negative controls from the DNA extraction and PCR steps used to identify contaminant sequences, independent aliquots of a bacterial mock community were processed together with samples to evaluate extraction, amplification and expected relative abundance of bacteria [36].

### Data processing and statistical analysis

DADA2 was used for exact sequence inference and chimera removal, followed by contaminant sequence removal using the ‘decontam’ R package [37]. Species-level taxonomic labels were assigned using BLASTn against the SILVA database (release 138). We assigned vaginal microbial community state types (CSTs) using the VALENCIA nearest centroid classification method for ease of comparing CSTs across studies [38]. For the purposes of our analysis, we combined CSTs IVA and IVB as CST IVA was relatively small.

Statistical analyses were performed using the ‘phyloseq’, ‘vegan’, ‘ImerTest’, ‘glmmTMB’, and ‘emmeans’ packages in the R statistical computing environment (version 4.1.3) [39-43]. Data were stratified by study visit and clinical variables of interest: HIV status, STI diagnosis at any time point, and pregnancy and birth outcomes. Shannon diversity and Bray-Curtis dissimilarity were used for analysis of alpha and beta diversity, respectively. Permutational multivariate analysis of variance (PERMANOVA) as implemented in the ‘adonis2’ R function was used to identify drivers of overall microbiome variation. Differences in CST composition and stability were assessed using a chi-squared test or Z-test of equal proportions as appropriate. Alpha diversity was compared using a mixed effects linear model with a subject-level random effect, and data are presented as estimated marginal means. Differential abundance testing at the species level was performed using a zero-inflated negative binomial model.

## Results

### Population characteristics

We recruited and enrolled 242 women, of which 107 (44%) were living with HIV (Table 1) The mean age was 29.5 years (SD 6.1), and 101 (42%) participants reported to be married or cohabitating. Most participants had completed secondary school (91%), and most were unemployed (61%). Mean gestational age at Visit A (first ANC visit) was 18.6 weeks (SD 6.3), with a range of 6–30 weeks (Supplement S1, Additional File 1). Gestational age range at Visit B (third trimester visit) was 24–36 weeks. Mean gestational age at the postpartum visit was 17 days after delivery (SD 28 days). The majority of women (98.8%) reported a single partner in the three months prior to Visit A.

Of the women living with HIV, 76 (71%) were already on ART at first ANC Visit (Visit A). A total of 80 participants (33%) tested positive for any STI at any time point, with 52 (22%) positive for CT, 14 (6%) positive for NG, and 38 (16%) positive for TV. The majority of STI diagnoses were made during Visit A (Supplement S2, Additional File 1). Overall, 90.9% of participants delivered full term, and 91.7% had live births without complications.

### Bacterial community composition

**Figure 1:**
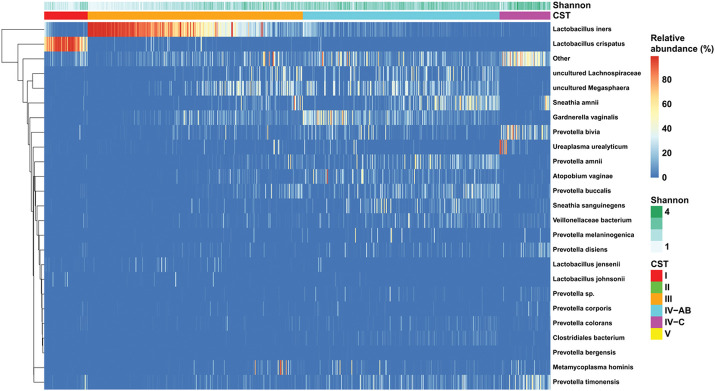
Heatmap of bacterial taxa identified by 16S sequencing of cervical swabs collected from 242 women across three Visits during pregnancy. CSTs determined by the VALENCIA classifier: CST I, dominated by *L. crispatus*; CST III dominated by *L. iners*; CST IVA, dominated by *Candidatus lachnovcurva vaginae* and *Gardnerella vaginalis* with moderate *Atopobium vaginae*; CST IVB, with high *G. vaginalis* and low *Ca. l. vaginae* and moderate *A. vaginae*; and CST IVC, *low lactobacillus, G. vaginalis*, and *A. vaginae* with high proportions of facultative anaerobes including *Prevotella* species. Shannon diversity index for each sample depicted across the top.

Based on a previously defined set of vaginal community types [38], we identified five CSTs: CST I, dominated by *L. crispatus*, CST III dominated by *L. iners*; CST IV-A, dominated by *Candidatus lachnovcurva vaginae* and *Gardnerella vaginalis* with moderate *Atopobium vaginae*, CST IV-B, with high *G. vaginalis* and low *Ca. l. vaginae* and moderated, *A. vaginae*; and CST IVC, *low lactobacillus, G. vaginalis*, and *A. vaginae* with high proportions of facultative anaerobes including *Prevotella* species (Supplement S3, Additional File 1). CST IV-A was relatively small and did not appear distinct from CST IV-B on the two major axes of the principal coordinates analysis (Supplement S3, Additional File 1), so these two were grouped into CST IV-AB for all remaining analyses (Fig. 1). The most prevalent CSTs were CST III (43% of all samples) and CST IV-AB (39% of all samples). Lactobacillus-dominant CSTs were the majority (51%), but only 9% of women were assigned to CST I. There were no samples assigned to CST II (*L. jensenii* dominant) or CST V (*L. gasseri* dominant). Approximately 10% of samples fell within CST IV-C, which was highly diverse and had large proportions of *Prevotella bivia, Prevotella timonensis*, and *Ureaplasma urealytica*.

### Changes in vaginal microbiota during pregnancy

**Figure 2:**
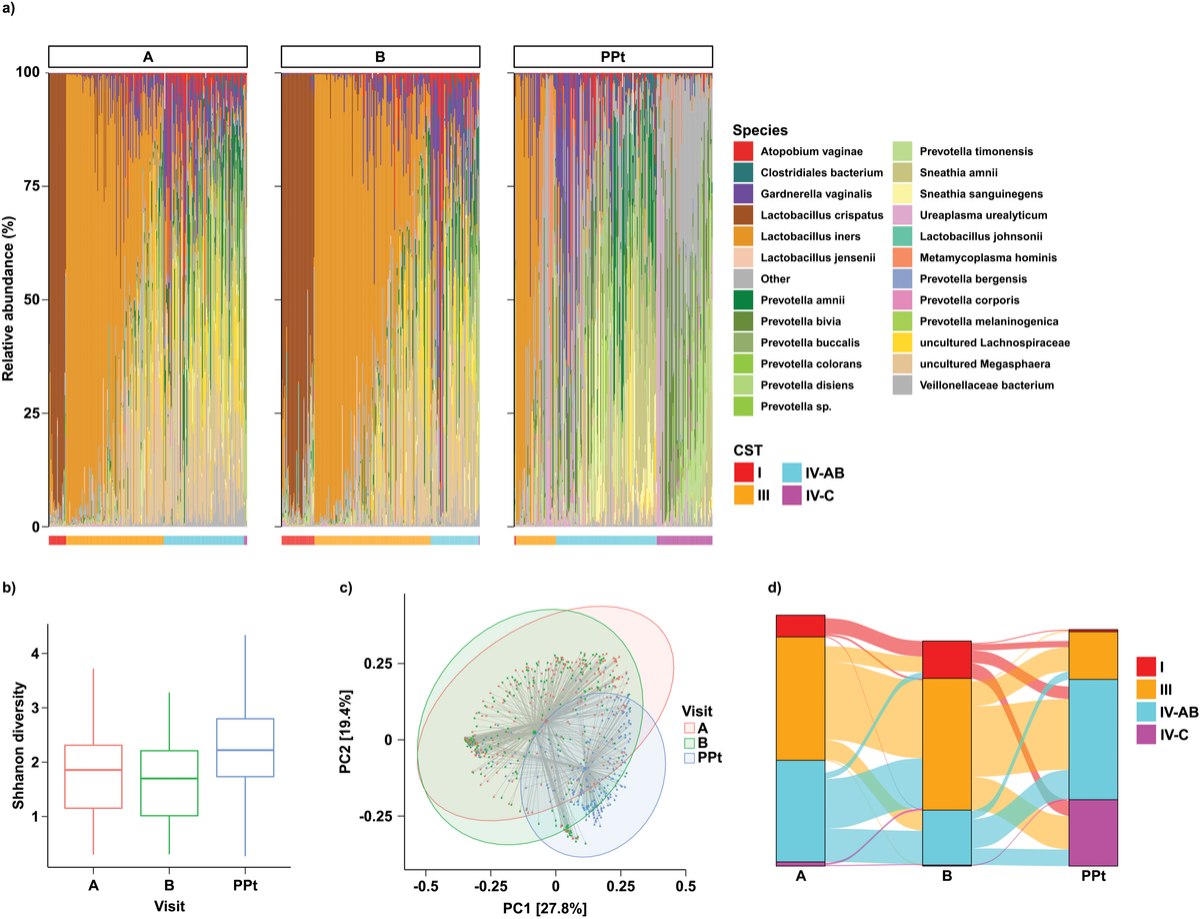
Composition of the vaginal microbiome across three visits during pregnancy. Visit A = initial visit (gestational age ranging from 6 – 30 weeks); Visit B = second trimester visit (GA 28-36 weeks); Visit PPt = postpartum visit (mean 17 days after delivery). (a) relative proportions of bacterial taxa at each visit across pregnancy. CSTs are shown across the bottom. (b) Shannon diversity between visits, presented as box plots with mean and interquartile range for each visit. Shannon diversity was higher at Visit PPt compared to Visit A (p = 2.0E-11) (c) PCoA of samples across visits using Bray-Curtis index (PERMANOVA R^2^ = 0.04, p < 0.001). Shaded ellipses denote 95% confidence intervals. (d) transitions between CSTs across visits. Moderate changes in CST distribution were observed from Visit A to B, with decreased CST IV-AB at Visit B, as a result of shifting to CST I or CST III. CST IV-AB was more likely to transition to CST III than to CST I (Chi-squared p < 1E-10). From Visit B to Visit PPt, a majority of CST III samples converting to CST IV-AB, while some members of CST I, CST III, and CST IV-AB all shifted to CST IV-C. CST III was more stable and less likely to transition than CST I (Chi-squared p = 0.048)

We next looked at the microbiome across Visits A (6–30 weeks), B (24–36 weeks), and postpartum (mean 17 days after delivery) (Fig. 2). Compared to Visit A, women at Visit B had higher relative abundances of *L. crispatus* and *L iners* and higher proportions of CST I and CST III (Fig. 2a). At the postpartum visit, women were more likely to have CST IV-AB and CST IV-C. Shannon diversity was significantly increased at the postpartum visit compared to Visit A (p < 0.001, Fig. 2b). Interestingly, very few samples belonged to CST IV-C at Visit A and Visit B, but a sizable proportion of CST IV-C emerged postpartum. Overall community composition differed significantly by visit or trimester (PERMANOVA R^2^ = 0.04, p < 0.001, Fig. 2c). Since Visit A varied widely in gestational age, we examined taxa plots from Visit A by week of gestation (Supplement S4, Additional File 1), which did not significantly differ. Moderate transitions in CST distribution were observed at an individual level from Visit A to B, with women with CST IV-AB at Visit A predominantly shifting to CST III (Fig. 2d, Table 2, Chi-squared p < 0.001).

During pregnancy, women with CSTs I and III had a more stable vaginal microbiota than those with IV-AB, whereas women with CST IV-AB more stable during the transition period from pregnancy to postpartum (Supplement S5, Additional File 1). From Visit B to postpartum, drastic CST transitions were observed, with a majority of CST III samples shifting to CST IV-AB, while a high proportion of women with CST I, CST III, and CST IV-AB all shifted to CST IV-C in similar proportions (Fig. 2d, Table 2). In this transition, very few women in both CST I and CST III remained in the same CST, but women in CST III were more likely than women in CST I to remain in the same CST at the postpartum visit (p = 0.048).

### Vaginal microbial associations with HIV serostatus

**Figure 3:**
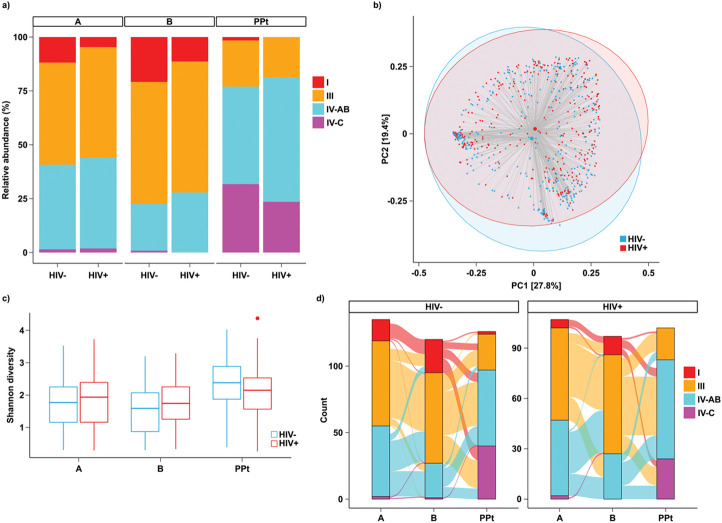
CST distribution across visits with respect to HIV status. (a) Relative proportions of CSTs across visits, stratified by HIV status (b) Shannon diversity at each visit by HIV status (c) PCoA by HIV status across all visits using Bray-Curtis index (PERMANOVA R^2^ = 0.002, p = 0.006). Shaded ellipses denote 95% confidence intervals. (d) transitions between CSTs across visits, stratified by HIV status. No differences in transition probabilities were observed.

We next assessed whether HIV infection and any STI diagnosis during pregnancy were associated with differences in the vaginal microbiome. CST distribution did not differ by HIV status at any visit (Fig. 3a, Table 3), although community composition differed significantly between women with and without HIV (PERMANOVA R^2^ = 0.002, p = 0.006, Fig. 3b). Shannon diversity was not significantly different between women with and without HIV (Fig. 3c). With respect to individual species, differential abundance testing showed that HIV infection was associated with lower relative abundance of *L. jensenii*at Visit B, and lower relative abundance of *P. corporis* and *bergensis* at the postpartum Visit, and higher *Metamycoplasma hominis* and *A. vaginae* at the postpartum Visit (Supplement S6, Additional File 1, p < 0.01). Transitions in CSTs did not appear to differ by HIV status (Fig. 3d). Specifically, there was no significant difference between women with and without HIV in the likelihood of transitioning from CST IV to CST I or III from Visit A to Visit B, and no significant difference in transition from CST I or III from Visit B to CST IV at Visit PPt, nor any differences in CST stability (Supplement S5, Additional File 1).

Overall, 29% of women living with HIV had not yet initiated ART at Visit A. A higher proportion of women living with HIV who had not yet initiated ART had vaginal microbiota characterized by CST IV-AB, though Shannon diversity was non-significantly higher in this group (Supplement S7, Additional File 1, unadjusted p = 0.06).

### Vaginal microbial associations with STI diagnosis during the peripartum period

**Figure 4:**
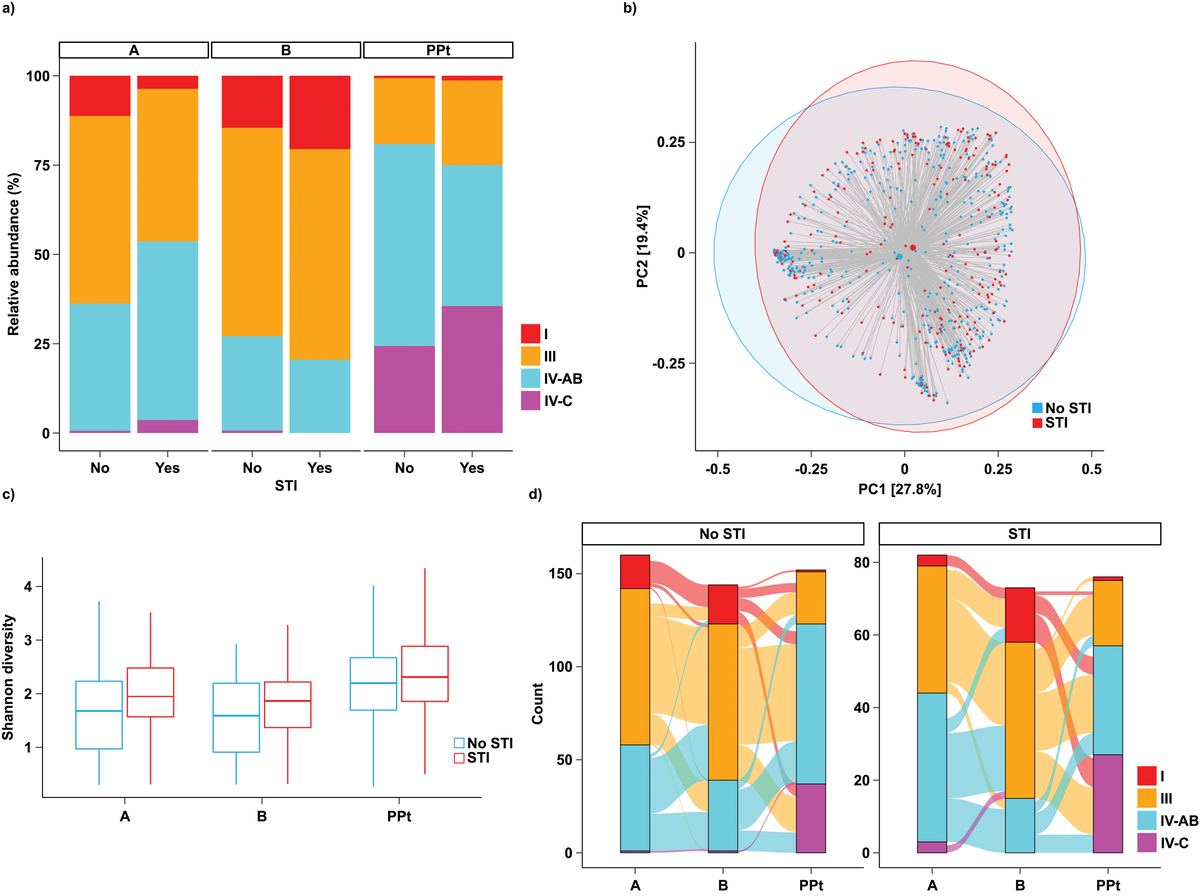
CST distribution across visits with respect to STI diagnosis at any visit (a) Relative proportions of CSTs across visits, stratified by STI diagnosis (b) Shannon diversity by STI diagnosis across all visits. At Visit A, Shannon diversity was higher among women who had been diagnosed with an STI (p = 0.004) (c) PCoA by STI diagnosis across all visits using Bray-Curtis index (PERMANOVA R^2^ = 0.002, p = 0.004). Shaded ellipses denote 95% confidence intervals. (d) transitions between CSTs across visits, stratified by STI diagnosis. No differences in transition probabilities were observed.

CST distribution differed between women with and without STI diagnosis (Fig. 4a). At Visit A, women with an STI diagnosis were more likely to be categorized as CST IV-AB or CST III (Fig. 4a, Table 3, Fisher’s exact p = 0.013). This pattern appeared to hold for each individual STI though did not reach significance for NG (Supplement S8, Table 3.). Only three women categorized as CST I at Visit A were diagnosed with STIs at any visit. Postpartum, there was a trend for women with an STI diagnosis to be more likely to have vaginal microbiota characterized as CST IV-C compared to any other CSTs (Table 3, p = 0.07).

Overall microbiome composition also differed between women with and without STI diagnosis ((PERMANOVA R^2^ = 0.002, p = 0.004, Fig. 4b). Shannon diversity was increased in women who had an STI diagnosis compared to women who did not at Visit A (Fig. 4c, p = 0.004) and at Visit B (Fig. 4c, p = 0.05). Individual taxa associated with STI diagnosis included *P. bivia, colorons, amnii and bucallis, Metamycoplasma hominis* and *Sneathia amnii*, at Visit A (Supplement S9, Additional File 1, p < 0.05), as well as *P. bucallis* at Visit B and other *Prevotella* species at Visit PPt (Supplement S9, Additional File 1, p < 0.05). Most of these associations appeared to be driven by *C trachomatis* (Supplement S8 and S9, Additional File 1). STI diagnosis did not appear to have any impact on CST stability and transition probabilities over the course of pregnancy (Fig. 4d, Supplement S5, Additional File 1).

### Vaginal microbial associations with adverse pregnancy and birth outcomes

In addition to HIV and STI status, we also examined birth outcomes and other variables for associations with microbiome composition. Overall 36 participants (15%) reported adverse pregnancy or birth outcomes including miscarriage, neonatal death, stillbirth, or preterm birth. We included these outcomes as a composite adverse birth or pregnancy outcome variable. CST distribution did not differ significantly between women who delivered full term live infants and women who had adverse pregnancy or birth outcomes (Supplement S10, Additional File 1). We did find a relationship between age and CST, with younger women being more likely to be in CST IV-C at both Visit A (p = 0.044) and Visit PPT (p = 0.035, Table 3).

## Discussion

We profiled the vaginal microbiome in a cohort of South African women over three visits during pregnancy and in the immediate postpartum period. We confirmed the transition diverse microbial signatures toward lactobacillus-dominant signatures during pregnancy, and identified the emergence of a facultative-anaerobe-rich, diverse signature in the early postpartum period which appears distinct from the vaginal microbial signatures of women early in pregnancy. We did not find a significant difference in CST distribution in women with and without HIV, but found that women diagnosed with STIs were more likely to have microbial signatures belonging to CST III or CST IV-A at first ANC visit. To our knowledge this is one of the first longitudinal studies of the vaginal microbiome in a cohort of pregnant South African women.

We categorized vaginal 16S rRNA gene sequences according to the Valencia nearest-centroid classification model for ease of comparison across populations. Consistent with prior studies among women of African descent [11, 20], about half of all samples belonged to lactobacillus-dominant CSTs, whereas half were dominated by diverse communities. Among the diverse communities, we found substantial populations of CST IV-AB and CST IV-C. A previous study in non-pregnant women from the FRESH cohort also identified two distinct populations of diverse communities, one dominated by *G. vaginalis*, and one dominated by facultative anaerobes including *Prevotella*. Other studies performed in African populations have also identified distinct anaerobe-dominant CSTs [15, 23, 27, 32], though this may vary with CST clustering methods [44] It is unclear whether these distinct CSTs are clinically significant, although in the FRESH cohort, the cervicotype dominated by facultative anaerobes showed a quadrupled risk of HIV acquisition versus the *Lactobacillus crispatus*-dominated CST, whereas the cervicotype dominated by *Gardnerella* did not after adjusting for diagnoses with CT.

During pregnancy, we noted a significant shift away from CST IV toward lactobacillus-dominant CSTs from early pregnancy to the third trimester, which is consistent with prior studies ([19, 24, 29, 30]. Serrano et al showed that American women of African descent had a decrease in diversity and transition to lactobacillus species early in pregnancy, as early as the second trimester [19]. Our data is limited by the variability of gestational age at first visit and the absence of a microbiome sample before pregnancy, but there was a clear shift toward lactobacillus species even into the third trimester (Visit B). Like Serrano et al, we noted that vaginal microbial profiles from CST IV were more likely to switch to CST III as compared to CST I [19].

In the postpartum period, we observed an increase in facultative anaerobic taxa and alpha diversity with a significant number of women falling into CST IV-C, which was not present in high proportions during pregnancy. We noted that both CST I and CST III were similarly likely to transition to CST IV-AB and CST IV-C postpartum. Unlike Goltsman et al, who found that CST I was more stable than CST III from the third trimester into the postpartum period, our data showed the opposite [45]. Previous studies have documented a sharp increase in diversity and anaerobic taxa during the postpartum period [28-30, 32], in part from a combination of decreased estrogen and changes in cervical remodeling during delivery [28]. Altogether, these results support a model by which pregnancy acts as a selective pressure toward a more optimal, Lactobacillus-dominant vaginal microbiome, possibly mediated by estrogen. Understanding the process by which this occurs may facilitate development of therapeutics, such as topical hormones, for beneficially altering the vaginal microbiome during or after pregnancy [46, 47].

We found differences in community composition, but not alpha diversity or CST distribution in the vaginal microbiota of women with and without HIV. Prior studies show increased alpha diversity in women living with HIV versus women without HIV [14, 16, 27]. However, some of these results may be confounded by ethnicity. Further, Price et al found that compared with women on ART, women not on ART have more diverse vaginal microbiomes with a higher proportion of anaerobes. Chehoud et al showed no difference in vaginal microbiota by HIV status when all women with HIV women were virally suppressed on ART [48]. In our study, a subset of women who were not on ART at the first ANC visit trended toward more CST IV-AB at Visit A compared to those established on ART, suggesting some of this discrepancy is explained by ART use or degree of immunodeficiency.

At the first ANC visit, women who were diagnosed with any STIs (CT, NG or TV) were more likely to be categorized as CST IV-AB or CST III than CST I. Our findings are consistent with previous studies noting fewer STI diagnoses in *L crispatus*-dominant profiles, and more STI diagnoses in both *L. iners*and anaerobe-dominant profiles [23, 49], and supports the hypothesis that *L. crispatus* is more protective against pathogens than *L. iners* and anaerobic species. More research is needed to understand whether and how *L. crispatus* protects against pathogens, and whether there are distinctions between *L. iners* and anaerobe-dominant microbiome profiles with respect to STI susceptibility, as STIs are a significant contributor to peripartum morbidity in sub Saharan Africa. We did not find any significant differences in the microbiome with respect to adverse birth outcomes including preterm birth. However our study was not powered to detect this difference and only 36 women in our sample had adverse pregnancy or birth outcomes.

Strengths of our study include its longitudinal design across pregnancy and into the postpartum period. In addition to the limitations already mentioned, others include small numbers of women living with HIV not on ART. For STI testing, as all women were treated, it is unclear if any subsequent changes in their microbiota were mediated by antibiotics. We did not collect data on inflammatory markers, metabolomics, or other vaginal biomarkers. Finally, our study took place among pregnant women at one ante-natal clinic in Cape Town, and results may not be generalizable across other regions or populations.

## Conclusions

Our study confirms a shift toward lactobacillus-dominance during pregnancy, and the rapid emergence of a distinct, highly diverse anaerobe-dominant vaginal microbial communities in the postpartum period. More work is needed to better understand the impact of the vaginal microbiome on perinatal outcomes and STI, HIV acquisition and vertical transmission. Researchers have suggested that the shift toward lactobacillus during pregnancy, which is mediated by estrogen and other physiologic changes, might foster a more “optimal” vaginal environment to prevent infection during pregnancy, which is then lost during the postpartum period. If this is the case, then there may be a role for hormonal and other therapies mimicking the physiologic changes of pregnancy to manipulate the vaginal microbiome toward an optimal state.

## Figures and Tables

**Table 1:** Characteristics of study participants (n = 242)

		n (%)
**Age (mean ± SD)**		29.5 ± 6.1
**Education**	Degree/Diploma	10 (4.1)
	Primary	11 (4.5)
	Secondary	221 (91.3)
**Employment**	Attending school/college	19 (7.9)
	Formal employment	73 (30.2)
	Informal employment	2 (0.8)
	Unemployed	148 (61.2)
**Gestational age at Visit A (initial antenatal visit, mean ± SD)**		18.6 ± 6.3
**HIV status**	Not living with HIV	135 (55.8)
	Living with HIV	107 (44.2)
**Already on ART at baseline**	No	31 (29.0)
	Yes	76 (71.0)
**Had vaginal sex during pregnancy**	No	17 (7.0)
	Yes	225 (93.0)
**Number of partners in last 3 months**	1	239 (98.8)
	2	3 (1.2)
**Any STI diagnosis at any visit**	No	162 (66.9)
	Yes	80 (33.1)
**CT diagnosis at any Visit**	No	190 (78.5)
	Yes	52 (21.5)
**NG diagnosis at any Visit**	No	228 (94.2)
	Yes	14 (5.8)
**TV diagnosis at any Visit**	No	204 (84.3)
	Yes	38 (15.7)
**Term delivery**	Full term	220 (90.9)
	Premature (born before 37 weeks)	22 (9.1)
**Pregnancy outcome**		6 (2.5)
	Live birth	222 (91.7)
	Miscarriage	7 (2.9)
	Neonatal death	2 (0.8)
	Still birth	4 (1.7)
	Termination	1 (0.4)

**Table 2:** Transitions across CSTs from: (a) Visit A (initial antenatal visit) to Visit B (third trimester) (b) Visit B (third trimester) to Visit PPt (postpartum visit)

(a)		To CST (at Visit B)			
		I	III	IV-AB	IV-C
From CST (at Visit A)*	I	15 (83.3%)	2 (11.1%)	1 (5.6%)	0 (0.0%)
	III	15 (13.6%)	75 (68.2%)	19 (17.3%)	1 (0.9%)
	IV-AB	6 (7.0%)	48 (55.8%)	32 (37.2%)	0 (0.0%)
	IV-C	0 (0.0%)	2 (66.7%)	1 (33.3%)	0 (0.0%)
					
(b)		To CST (at Visit PPt)			
		I	III	IV-AB	IV-C
From CST (at Visit B) ^#^^	I	1 (2.9%)	6 (17.6%)	12 (35.3%)	15 (44.1%)
	III	1 (0.8%)	23 (18.5%)	68 (54.8%)	32 (25.8%)
	IV-AB	0 (0.0%)	8 (15.4%)	28 (53.8%)	16 (30.8%)
	IV-C	0 (0.0%)	0 (0.0%)	1 (100.0%)	0 (0.0%)

* Women in CST IV at Visit A were more likely to transition to CST III than CST I at Visit B (Chi-squared p < 0.001).

# Compared to CST III, women in CST I at Visit B were less likely to remain in the same CST at Visit Ppt (Chi-squared p = 0.048)

^ At Visit B, there was no significant difference in the likelihoods of women of CSTs I, III, or IV–AB to transition to IV–C at Visit Ppt (p=0.1177)

**Table 3:** Differences in participant characteristics across CSTs at Visit A (n = 242)

		CST				P- value
		I	III	IV-AB	IV-C	
Median age (IQR)		32 (26–37)	29 (24–35)	30 (26–33)	22.5 (20.5–24.5)	0.044
Having vaginal sex at Visit A	Yes	20 (95.2%)	112 (94.1%)	89 (90.8%)	4 (100%)	0.77
	No	1 (4.8%)	7 (5.9%)	9 (9.2%)	0 (0%)	
HIV/ART status	Not living with HIV at baseline	16 (76.2%)	64 (53.8%)	53 (54.1%)	2 (50%)	0.256
	Living with HIV, not on ART at baseline	2 (9.5%)	12 (10.1%)	16 (16.3%)	1 (25%)	
	Living with HIV and on ART at baseline	3 (14.3%)	43 (36.1%)	29 (29.6%)	1 (25%)	
STI diagnosis at any visit	Yes	3 (14.3%)	35 (29.4%)	41 (41.8%)	3 (75%)	0.013
	No	18 (85.7%)	84 (70.6%)	57 (58.2%)	1 (25%)	
CT diagnosis at any visit	Yes	1 (4.8%)	21 (17.6%)	30 (30.6%)	1 (25%)	0.019
	No	20 (95.2%)	98 (82.4%)	68 (69.4%)	3 (75%)	
NG diagnosis at any visit	Yes	1 (4.8%)	7 (5.9%)	7 (7.1%)	2 (50%)	0.068
	No	20 (95.2%)	112 (94.1%)	91 (92.9%)	2 (50%)	
TV diagnosis at any Visit	Yes	2 (9.5%)	18 (15.1%)	14 (14.3%)	3 (75%)	0.037
	No	19 (90.5%)	101 (84.9%)	84 (85.7%)	1 (25%)	
Birth outcome	Normal	18 (85.7%)	94 (79%)	72 (73.5%)	3 (75%)	0.794
	Adverse event	3 (14.3%)	24 (20.2%)	24 (24.5%)	1 (25%)	
	Missing outcome	0 (0%)	1 (0.8%)	2 (2%)	0 (0%)	

## Abbreviations

ART: anti-retroviral therapy
CST: community state type
CT: *Chlamydia trachomatis*
G: grams
HIV: human immunodeficiency virus
NG: *Neisseria gonorrhea*
STI: sexually transmitted infection
TV: T*richomonas vaginalis*
